# ‘Making every contact count’ with patients with musculoskeletal conditions: a qualitative exploration of acceptability to physiotherapists

**DOI:** 10.1186/s12913-023-10126-1

**Published:** 2023-10-19

**Authors:** Amelia Parchment, Wendy Lawrence, Em Rahman, Nick Townsend, Elaine Wainwright, David Wainwright

**Affiliations:** 1 Department for Health, University of Bath, Bath, BA2 7AY England, UK; 2 NIHR Applied Research Collaboration- Greater Manchester, University of Manchester, Manchester, M13 9PL England; 3 Primary Care, Population Science and Medical Education, Faculty of Medicine, University of Southampton, Southampton, SO16 6YD England, UK; 4 Public Health Workforce Development, Southern House, Health Education England, Winchester, SO21 2RU England, UK; 5 Centre for Exercise, Nutrition and Health Sciences, School for Policy Studies, University of Bristol, Bristol, BS8 1TZ England, UK; 6 Aberdeen Centre for Arthritis and Musculoskeletal Health (Epidemiology Group), School of Medicine, Medical Sciences and Nutrition, University of Aberdeen, Aberdeen, AB25 2ZD UK

**Keywords:** Making every contact count, Healthy conversation skills, Physiotherapy care, Behaviour change, Prevention, Self-management

## Abstract

**Aim:**

To qualitatively explore physiotherapists’ experiences and acceptability of implementing ‘Making Every Contact Count Healthy Conversation Skills’ (MECC HCS) in routine practice with patients with musculoskeletal (MSK) conditions.

**Methods:**

This article reports the second phase of a mixed method, sequential explanatory study. Physiotherapists trained in and delivering MECC HCS in their practice were invited to take part in semi-structured interviews. We hoped to develop a rich, in-depth understanding of their use and perceptions of the brief intervention and to contextualise findings from the first phase of the study. Qualitative data were analysed using Reflexive Thematic Analysis.

**Results:**

Physiotherapists valued MECC HCS as being integral to their practice, promoting a person-centred approach to supporting behaviour change and enhancing self-management in patients with MSK conditions and pain. It was believed that this brief intervention could reduce workload pressure for physiotherapists and have significant wider benefits for health services. Time limitations associated with appointments did, however, pose as a challenge to MECC HCS delivery, and it was felt that more organisational-level support was needed to sustain it.

**Conclusions:**

These findings support our quantitative data, collected in the first phase of this study. MECC HCS is a highly acceptable brief intervention that can be delivered in physiotherapy care to support behaviour change in patients with MSK conditions. Future roll-out may be optimised within organisations by providing regular refresher training and adopting a MECC champion.

**Supplementary Information:**

The online version contains supplementary material available at 10.1186/s12913-023-10126-1.

## Introduction

Musculoskeletal (MSK) conditions are a rising public health concern and a leading contributor to the total burden of ill health globally [1, 2]. Although diversity exists between pathophysiology and diagnoses, pain and impaired physical functioning are unifying characteristics of MSK conditions, and primary mechanisms leading to disability and loss of work [3–7]. An ageing population and increase in prevalence of risk factors for non-communicable diseases are predicted to contribute to a steep rise in the number of people living with MSK conditions and pain [1, 8]. Some modifiable risk factors, such as stress, physical inactivity, smoking and obesity are additionally associated with [9–11] these conditions and their symptoms being chronic (lasting longer than three months). Those living with chronic MSK conditions and pain score lower on quality-of-life measures, are four times more likely to experience depression, and are less likely to be in employment than those without a long-term condition [2]. Many also live with multimorbid conditions, such as hypertension and cardiovascular disease [12, 13]. The clear impact on the individual, their employers, health and care services and the wider economy warrants effective public health initiatives for the prevention of chronic MSK conditions and the promotion of good MSK health.

The scaling up of evidence-based behaviour change interventions in routine healthcare practice is one vision of Public Health England for improving population MSK health and reducing the prevalence of those living with pain and disability [14]. This aligns with guidance from National Institute of Clinical Excellence [15, 16], which proposed the integration of different intensities of behaviour change intervention in healthcare settings to target risk factors associated with chronic, non-communicable diseases. These range from very brief interventions, which can be delivered by anybody in direct contact with the public, to high intensity interventions, delivered by specialists in behaviour change. NICE provides evidence showing that the delivery of even very brief or brief interventions (V/Bis), lasting from under a minute to around fifteen minutes, is effective and cost-effective in supporting behaviour change relating to smoking, alcohol use, physical activity and diet in patient populations [15–18].

People with MSK conditions and related pain are the largest patient group treated in physiotherapy services [19]. Moreover, contacts between patients and physiotherapists are increasing with the roll-out of first-contact practitioners (FCPs), facilitating immediate access to physiotherapy expertise for patients presenting with MSK complaints whilst reducing demand on general practice and secondary care [20]. These roles have been well received, with physiotherapists reporting positive experiences of working in FCP services and patients reporting very high satisfaction with FCP consultations [21]. Physiotherapists and FCPs are therefore uniquely placed to support behaviour change and promote health using very brief or brief interventions with these patients. However, whilst they have been found to perceive health promotion positively and as part of their role [22], physiotherapists have previously missed opportunities for, and showed a lack of understanding of V/BIs [23].

The introduction of a commitment to ‘Making Every Contact Count’ (MECC) in standard NHS contracts has meant that individual trusts and organisations have had to establish ways in which they can upskill staff to routinely, and opportunistically, deliver V/BIs. MECC is an evidence-based intervention, drawing on the Capability, Opportunity and Motivation Behaviour (COM-B) model [24] which posits that these three constructs interact to influence behaviour and was developed in response to the NICE behaviour change guidance [15]. It utilises the thousands of interactions had by frontline workers with the public to promote health, with the aim of embedding prevention into practice [25]. Approaches to training and delivery of MECC differ [26–28] and variations in implementation success have been reported [29]; however, data from local evaluations within MSK services have been promising. Physiotherapists report an increase in their confidence in supporting behaviour change with their patients, post- MECC training [30] and services have seen an increase in discussion around health risk factors during physiotherapy appointments after upskilling staff in MECC V/BIs [31]. Moreover, referral data suggest an increase in patients completing supportive sessions for healthy eating, weight loss, smoking cessation, and mental health, following the uptake of holistic, MECC consultations [32]. Despite this, there are discrepancies between the number of patients believed to benefit from V/BIs and the number of patients to which MECC is actually delivered [33, 34].

Empirical research aiming to understand factors that might influence how and why MECC is (or is not) adopted in physiotherapy care, as recommended, is scarce. Known as ‘implementation science’, this type of research is important for increasing understanding in how to bridge the gap between care that is effective and care that is delivered, in order to achieve desired change [35, 36]. In turn, this could optimise the future roll out of interventions, improve strategies, and sustain these strategies over time and in new contexts [37]. Handley et al. [38] highlight the fundamental principles in implementation science including: (a) the use of behaviour change theory to explore determinants of current behaviour and how to achieve desired change; (b) directly engaging with targeted individuals and stakeholders, in order to increase usefulness and applicability of findings for intended groups; and (c) approaching research in a flexible, non-linear way, reflecting the changes that can occur in real-life, applied settings and the influence these can have on implementation. This paper describes part two of a mixed-methods study, which drew upon implementation science approaches, and aimed to evaluate the uptake, perceptions, and acceptability of the Wessex model of MECC for physiotherapists supporting people with MSK conditions. Participants had received formal training in this over one year prior to the study period.

**Fig. 1:**
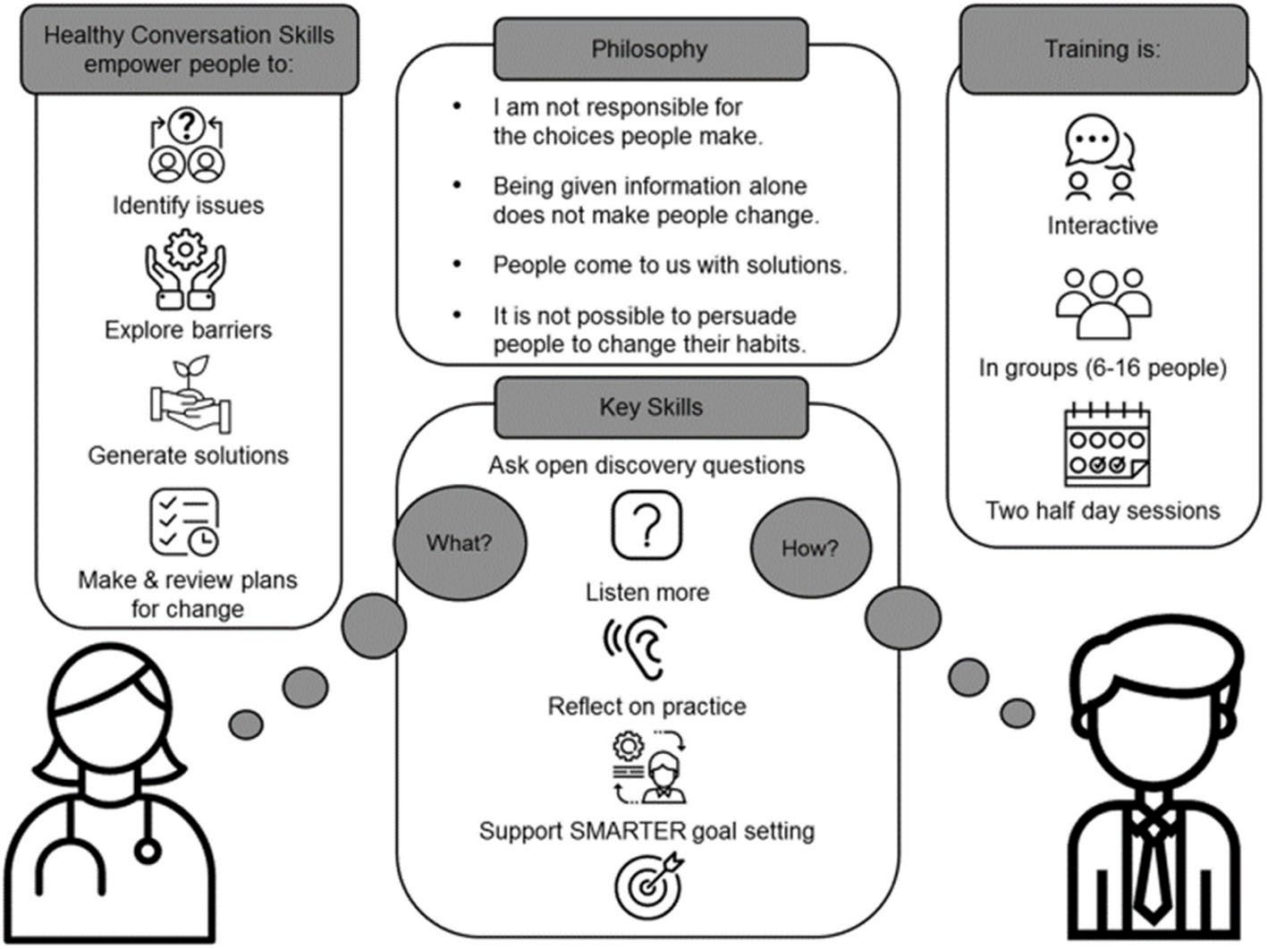
Healthy Conversation Skills philosophy, skills and training delivery [54]. Note. SMARTER: Specific, Measurable, Action-oriented, Realistic, Timed, Evaluated, Reviewed

The Wessex model of MECC incorporates Healthy Conversation Skills (HCS); an empowering, person-centred approach to behaviour change [39, 40] which recognises that giving information alone is not sufficient to change behaviour. Rather, individuals must feel able and motivated to change [41, 42]. Trained practitioners have demonstrated improved competence and confidence in supporting behaviour change up to one-year post-training, and service users have been found to feel more satisfied with their care and make more positive changes than those not receiving the intervention [43, 44]. Based on Social Cognitive Theory [45] and underpinned by the Taxonomy of Behaviour Change Techniques (BCTs) [46], training aims to build the self-efficacy of practitioners in addressing health behaviours and supporting patients to change (Fig. 1). Skills are developed to understand the world and context of the patient, help patients find their own solutions to issues and identify first steps to change, without adding to overall appointment time. Patients are thus empowered to take control of their behaviours, in turn, increasing their self-efficacy; a construct strongly associated with more healthful behaviours [47, 48]. Skills can also be used to have empowering, person-centred conversations even if immediate change is not the priority. A person-centred approach encourages a holistic, biopsychosocial model of care, shared decision making and a therapeutic alliance [49]. For physiotherapists supporting individuals with MSK conditions and pain, this has been recommended in high quality clinical practice guidelines [50], and is considered highly important by patients themselves [51–53].

This approach has also been supported by physiotherapists. Those that are trained in MECC Healthy Conversation Skills and supporting people living with MSK conditions and pain scored the intervention as highly acceptable, appropriate, and feasible within their role, and reported using their skills daily to support behaviour change (as observed in part one of this study, which is described elsewhere [34]. Perceived organisational capacity and resources for sustaining it was, however, found to be only moderate, and there were missed opportunities for delivery of the brief intervention in practice [34]. The aim of the present paper was to (a) contextualise these findings further, by gaining an in-depth, qualitative understanding of the views, experiences, and acceptability of MECC Healthy Conversation Skills for the physiotherapists and, (b) discuss these qualitative findings in relation to behaviour change theory in order to provide recommendations for future implementation.

## Methods

### Ethics

This study recruited NHS staff only, and therefore did not require approval from the local NHS Research Ethics Committee. The study did, however, receive necessary approvals from the Health Research Authority (HRA), reference 20/HRA/2919, and University of Bath’s Research Ethics Approval Committee, reference EP19/20,057. All participants provided electronic informed consent prior to the interview taking place, via ‘Online Surveys’.

### Design

This paper describes findings from the second phase of a mixed methods, sequential explanatory study. The first phase is reported elsewhere [34] and quantitatively explored, using an online survey, the use and perceptions of MECC HCS as a brief intervention across professional groups in the UK. This generated the purposive sample for the present, qualitative phase. The purpose of collecting these qualitative data, via semi-structured interviews, was to gain a deeper understanding of physiotherapists’ views on the acceptability of using MECC HCS to support people living with MSK conditions to change their behaviour. Further detail regarding the integration of quantitative and qualitative components of this study have been reported elsewhere as part of a PhD thesis and can be requested from the lead author.

This study was underpinned by critical realism, which recognises that whilst an objective, universal reality might exist, this cannot be accessed by the researcher. Rather, only subjective, situated perceptions and interpretations of reality can be studied, and shape what is known about the world [55]. This subjectivity relates not only to the perspectives of participants involved in research but also the researchers who construct the findings [56]. An understanding of both context within which experiences occur, and the influence of the researcher is therefore important. This critical realist approach informed the use of reflexive thematic analysis to provide an interpretation of the data.

### Participants

Physiotherapists of all levels of experience were eligible for inclusion in this phase of the study, as long as they were working with patients living with MSK conditions and/or pain. All participants were required to have been trained in and have experience of delivering MECC HCS as a brief intervention to patients within the NHS. All were using HCS in practice at least daily and had attended MECC HCS training at least one year ago, as self-reported on the online survey.

Information power was used to determine the point at which data collection stopped [57], as were the recommendations of Braun and Clarke [58] for a study of this size and scope (10–20 participants). The former approach to justifying sample size (as opposed to ‘saturation’) is encouraged for those using reflexive thematic analysis, due to the subjective role of the researcher in generating meaning through interpretation, not excavation, of data [59].

### Procedure

In the online survey (mentioned above), participants were asked to consent to be contacted for follow up research and to share contact information. Physiotherapists supporting people living with MSK conditions were identified from these data and contacted by the lead researcher to discuss participation in an interview. Those who were interested in participating were sent an online consent form (including a link to the participant information sheet). Electronic informed consent was obtained from all participants before they took part in virtual interviews.

Following a semi-structured interview guide (Appendix 1), the researcher asked exploratory, open questions to gain a deeper understanding of the physiotherapists’ experiences and perceived acceptability of implementing MECC HCS in routine practice. This interview guide had been piloted with a healthcare professional who was not involved in this research, to gauge whether the proposed interview questions were clear and could prompt qualitative data that would adequately address the study’s research questions. No modifications were made to the guide. Some questions were informed by the COM-B model of behaviour [24] and explored participants’ capability, opportunity, and motivation to implement MECC HCS. According to the model, these components interact to influence behaviour, and have been a focus throughout this study for understanding factors that could influence physiotherapists’ uptake of MECC HCS.

Interviews took place between November 2020 and April 2021 and were conducted virtually using Microsoft Teams. All interviews were audio-recorded, transcribed verbatim and anonymised at the point of transcription. They lasted, on average, 33 min and were conducted by a single researcher (AP).

### Data analysis

Following Braun and Clarke’s [60] six main stages, reflexive thematic analysis was employed both inductively and deductively to identify patterns of meaning in the qualitative interview data. Detailed notes and records were kept throughout the analytical process.

The lead researcher (A.P.) first familiarised herself with the data through reading and rereading the transcripts several times. Next, she developed initial codes, highlighting patterns of meaning within the data using Microsoft Word. A predominantly inductive, ‘data driven’ approach was taken at this stage, whereby the dataset was used as the starting point for engaging with meaning [61]. An element of deductive analysis did, however, ensure that coding was relevant to the research topic, and at times the COM-B model of behaviour [24] was used for interpreting meaning. Both semantic and latent coding were utilised. Codes were therefore developed using the explicit meanings of the data, communicated by the participants (semantic coding) and deeper levels of meaning, interpreted by the researcher (latent coding) Initial themes were then generated using these codes. Themes were reviewed and refined in an iterative manner, and an initial thematic map was developed. AP then met with E.W. to discuss the thematic map, codes and themes. The role of E.W. as a ‘critical friend’ was to enhance critical reflection of A.P.’s involvement/subjectivity in the analysis (reflexivity), and interpretation of the data, rather than to reach an objective consensus about theme development. The lead researcher was, for example, a 26-year-old PhD researcher who had not experienced chronic pain herself but was personally invested in this topic due to having relatives and friends who did experience chronic pain. Engaging with a ‘critical friend’ was considered an appropriate approach to reflexive thematic analysis, given that reflexivity and acknowledging and understanding one’s subjectivity in data collection and analysis are important tools for this particular qualitative method [60]. This enabled the final naming and defining of themes before write-up began.

## Results

Of the 15 eligible participants who completed the online survey in the first phase of this study [34], 11 consented to take part in follow up interviews (73%). Given the study aim, specificity of characteristics among the participants, theoretical background of the research and quality of dialogue in the interviews, we believed sufficient information power was reached in order to answer the study research questions [57]. Participants were aged between 29 and 53 (M = 41.8, SD = 8.4) and 9 were female (82%). Participants were mostly physiotherapists currently working with patients with MSK conditions and related pain (10/11, 91%). One (9%) had been promoted from their previous physiotherapy role and now worked as head of therapies with reduced patient contact. Four worked within NHS trust based in Hampshire (36%), two within Dorset (18%), two within Devon (18%), one within Isle of Wight (9%), one within Bath and North-East Somerset (9%), and one worked across NHS sites in Dorset, Hampshire and Wiltshire (9%).

All had completed at least MECC HCS ‘Lite’ (a condensed version of the full MECC HCS programme, consisting of 1 × 3 h training session), two had completed the full MECC HCS training programme (2 × 3 h training sessions) (18%) and three had completed the MECC HCS Train the Trainer programme (27%).

### Themes

Five main themes were developed during Reflexive Thematic Analysis: ‘Recognising the patient as the expert supports change’, ‘MECC HCS improves physiotherapy practice’, ‘MECC HCS shared problem solving reduces workload’, ‘time as a perceived barrier to MECC HCS’ and ‘system-level support needed to sustain MECC HCS’. These themes and their subthemes are outlined below with illustrative quotes.

### Theme 1: recognising the patient as the expert supports change

This theme encompasses how participants perceived viewing patients as the experts in their own lives important for enabling change. It highlights perceptions of MECC HCS as promoting an effective, person-centred approach to physiotherapy care that can support behaviour change, but also an approach that is holistic, taking into consideration the realities of personal barriers to change. Participants felt that acknowledging and addressing these barriers could promote future change, if this was not possible in the here and now.

#### Subtheme 1: a holistic, person-centred approach enables change

Participants highlighted how adopting a person-centred approach during physiotherapy appointments, through the use of MECC HCS, was important for “*allowing the patient to speak and be heard*” [Participant 11], thus, engaging them in their own physiotherapy care: *“If you have just asked them robotic questions and not taken into consideration their lifestyle and wellbeing, you’re not making them count and therefore they’re not going to be as onboard with you and what you’re trying to achieve with them”* [Participant 2]. They described that opening the discussion up in this way, *“seeing the patient as not just the isolated problem”* [Participant 3] and valuing them as the expert in their own world allows the physiotherapist to learn more about the lives of their patients, and support behaviour change in an empowering way: *“I actually learn a lot more with the patients, and I’ve learnt to value their knowledge about their condition and how they’re coping… for that two minute conversation or even less, you can plant the seeds for the patient to reflect about their own way of living, about their own potentials and their own talents and skills, so what they can put into practice, making them more aware and more empowered”* [Participant 8]. This was an approach to physiotherapy care that was recognised as being different, but more positive and supportive, than what was previously considered the norm: *“Twenty years ago, it was very much about you being right and telling people what they needed to do and, you know, it’s now developing those skills of how do you actually encourage people to change rather than just telling them what my opinion is and what I think you should do?”* [Participant 10].

#### Subtheme 2: acknowledging personal barriers enables future change

It was, however, recognised by many participants that change was not always possible for patients in the present moment, and that they may face a variety of barriers, personal to them and their situation, that could prevent them from making beneficial behaviour changes. Acknowledging and addressing these personal barriers with the patient, with the use of open discovery questions, was considered a steppingstone to the possibility of them thinking about future behaviour change: *“Recently I had a lady who had early arthritis in her knee. She was struggling and was already suggesting to me that she hasn’t got much motivation to think about lifestyle change. I could just use a couple of open discovery questions with her to open up and I don’t think we would action anything at that point in time… but it made her think of her options so she could go away and think about it”* [Participant 9]. Participants emphasised that by at least *“planting the seed”* in this way, after recognising that a patient is not ready or able to change right now, means that “*future behaviour change is still possible*” [Participant 5]. It was also recognised that even those in the process of change may face personal challenges that could impact progress. Participants highlighted that flexibility when delivering MECC HCS is beneficial for supporting behaviour change in this scenario : *“We know that we should make things [goals] SMART but we will just try and sort of chip away at it because the mindset of somebody on one appointment may be very different to the next time they come in and other things may have happened that may affect their mindset, so the goals are flexible…we just sort of document that”* [Participant 6].

### Theme 2: MECC HCS improves physiotherapy practice

The delivery of MECC HCS was supported within the physiotherapy role by all participants. Many felt that using opportunities to discuss lifestyle factors and their impact on MSK health was an already integrated, important part of care. It was, however, highlighted that training in MECC HCS developed the skills needed to have effective, person-centred conversations to support behaviour change with MSK patients. These conversation skills were considered particularly important for topics perceived as being more difficult to address, such as weight. Participants therefore recommended offering MECC HCS training to physiotherapy students, at the very beginning of their careers, to increase confidence in having health-related conversations and to ensure these skills are embedded in practice.

#### Subtheme 1: the principles of MECC HCS are already ingrained

The majority of participants described MECC was a philosophy that was already integrated into much of their routine physiotherapy care: *“I’d say a good 70% of our time is around trying to change health beliefs, health attitudes. Lifestyle is a huge part of our caseload. A really small part is gaining information and the rest is about their lifestyle management with their condition, generally”* [Participant 11]. This was deemed an essential part of care, which could prevent future regression in the health of the patient: *“MECC has to be part of treatment. I don’t think you can treat effectively otherwise because people will just go back to whatever poor health behaviours they may have afterwards and then the problems return”* [Participant 1]. One participant, who worked as a First Contact Practitioner within a GP surgery, highlighted how she felt physiotherapists *“look at the bigger picture”* [Participant 9] when treating patients. She emphasised that her and her physiotherapy colleagues are generally *“more biopsychosocial”*, enabling holistic, health-related conversations. In contrast, she described the general practitioners she works alongside as adopting an approach that is *“almost always medical”*, with *“no thinking about the wider picture”*. Some participants felt that they had a personal role in their patient’s health and wellbeing, and that the use of relevant open discovery questions was common practice: *“Every single patient I meet I have a role in their general wellbeing. So, every single patient I meet, and I think every physio will tell you this, they ask about your lifestyle, so ‘what do you do for work? What are your sports, hobbies? What kind of thing would you do to keep yourself happy and fit?’…”* [Participant 2].

#### Subtheme 2: MECC HCS facilitates difficult conversations

However, despite perceiving MECC as already being part of routine practice, receiving formal training highlighted that there was room for improvement in having healthy conversations with patients: *“How I felt initially when I went on the training was that ‘I’m sure I do this already’, and I do but I just didn’t really expand or explore it properly”* [Participant 9]. In support of this, participants reported MECC HCS training as developing the skills physiotherapists needed to have more challenging conversations with patients regarding risk factors affecting their MSK health: *“Some conversations aren’t easy to have, particularly around weight loss or increasing exercise, which is generally the conversations we have. So, giving staff the confidence and skills to go and have those conversations or ask those questions to patients which, when you’re newly qualified, it’s more challenging to do… MECC helps with that”* [Participant 11]. Others highlighted that more practice made these conversations easier, with the use of open discovery questions enabling the patients to identify their own health-related issues: *“Whenever you’re discussing it [weight] in a physio context… it’s saying, ‘what are the health-related problems that your weight could be causing to you?’ I think it is quite a difficult subject to bring up, but I think the more I did it, the easier I found it was for them to tell me that it was the problem rather than me to tell them”* [Participant 7]. It was suggested that further support could then be offered to target these problems. Participants also recognised that MECC could *“go beyond just how lifestyle affects your knee pain”* and the intervention *“offered permission”* for addressing risk factors for other, comorbid conditions [Participant 10], therefore providing a method of comprehensive health promotion.

#### Subtheme 3: physiotherapy students should be MECC HCS trained

Many felt that the relevance of MECC in physiotherapy practice was so high, that training student physiotherapists was essential, increasing the likelihood of healthy conversation skills being embedded upon starting their career: *“We need staff coming through who’ve got that skillset, that qualification so that it’s embedded into their practice… I think if we could get [MECC] ingrained via undergraduate programmes to that particularly in musculoskeletal, but also on the wards, you would maybe find clinicians that are coming readymade with that at the forefront of their agenda… targeting undergraduate programmes would be key”* [Participant 4]. It was suggested that training at this level would equip new starters with the confidence to have healthy, open conversations with patients; a skill that was emphasised as being highly important in practice: *“I think how to have a proper conversation is as important as knowing all the clinical stuff and doing all the treatment so I think it needs to be brought in at undergraduate level to allow people the confidence to have those open ended conversations”* [Participant 1]. One participant reported the university in which she works as being the first to roll MECC HCS to undergraduate students and regarded the first-year students receiving this training as *“lucky to have it”*. She described her own experience of becoming accustomed to a particular way of working within her role over a number of years and emphasised that utilising MECC earlier on in her career could have been significantly beneficial: *“I was trained, like I said, in the ‘90s, where you tell patients what to do. You fix their problems. So, I have already had my own bias and my own ingrained way of ‘this is how we work as physios’, for example. If I was taught [MECC] from first year [undergrad] then God knows where I’d be now, if I’d been using MECC for the last 23 years”* [Participant 8].

### Theme 3: MECC HCS shared problem solving reduces workload

Theme three encompasses how physiotherapists viewed MECC HCS as encouraging patients to self-manage, with potential to increase health-related independence and reduce contact with health services. Empowering patients to take control of their health and building their self-efficacy to self-manage was also felt to reduce pressure experienced by practitioners to fix patients’ problems. Long-term prevention through the delivery of MECC HCS was envisioned by participants. This was believed to have potentially significant benefits for the health service, including reduced admissions and cost savings.

#### Subtheme 1: promoting self-management

Participants highlighted how MECC HCS facilitates shared decision making with patients, reducing feelings of pressure for practitioners to *“fix the patient’s problem”* and instead supporting patient self-management: *“This way of having a conversation and engagement with the patient, I feel like the responsibility is shared which makes me feel less stressed and pressured… I’m more confident with my communication skills in terms of making sure that I facilitate, you know, empowering patients and promoting self-management”* [Participant 8]. This self-management was considered important for improving future health outcomes: *“When we’re seeing people with lower limb weight bearing joint pain… especially arthritis type conditions, there’s specific exercises you can give people but really it’s about a lifestyle modification that’s going to make a long-term difference and I think we see repeated service users that were not embedding lifestyle changes to enable them to be independent… When you’ve got a receptive patient, [MECC HCS] can make a really big difference for how many times we have to see them, but also for them changing their life and having an improvement in it as well”* [Participant 7]. It was emphasised that, despite it being relatively well known that one can be directly responsible for many elements of their own health, *“self-efficacy that patients have for that is very low”* [Participant 4]. Supportive, healthy conversations were therefore considered a priority in clinical practice for supporting self-management and could support effective change in patients, even when this first appears difficult to do.: *“In reality, we know [having these conversations] are the most useful rather than any of their exercises or manual treatment we do. Giving someone confidence and reassurance is the key to their management and moving forward”* [Participant 11].

#### Subtheme 2: promoting prevention

The delivery of MECC HCS was additionally considered an important steppingstone for long-term prevention for those with MSK conditions, reducing the likelihood of them needing future MSK healthcare intervention: *“If we can improve people’s lifestyles early on it might prevent them needing orthopaedic intervention like, you know, further down the line”* [Participant 7]. Participants additionally emphasised this prevention in relation to comorbid conditions, which require access to other specialist services: “*Thinking about MSK, that if we eat healthily and if we reduce our weight and do all of that sort of stuff, you know, your chances of developing diabetes are significantly reduced, hypertension significantly reduced and therefore you can then sort of progress that onto thinking about the wider effects of the health service if you’ve got fewer people requiring diabetes services and such”* [Participant 6]. Focusing on prevention via MECC HCS was highlighted as beneficial for both the economy: *“It is a huge cost saving if we can help change the health beliefs and behaviours of our patients. It decreases comorbidities, any admissions, just accessing healthcare particularly at the moment, then obviously economic benefits to workforces, keeping people in work in some shape or form”* [Participant 11] and the patients: *“ [MECC HCS] can help improve the health of patients, you know, as a whole, in the whole nation… we should be doing everything we can to try and support people to better themselves and give them a better quality of health and everything”* [Participant 3].

### Theme 4: time as a perceived barrier to MECC HCS

Time was considered a significant barrier to participants implementing MECC. This related to both time taken to engage in formal training in order to develop necessary skills, and time in routine appointments for delivering the brief intervention. Interestingly, others felt that it was a misperception that MECC requires additional time to implement during physiotherapy appointments. Rather, these participants believed that practice helped to embed skills into routine care and within usual appointment times. This approach was felt to have become a natural way of engaging with patients, rather than an extra duty.

#### Subtheme 1: time limitations associated with the role

Taking time out from the clinical role to attend formal training in MECC was highlighted as a main barrier to implementation: *“If I’m taking time out to do some training, then obviously your day job doesn’t go anywhere… time and funding are the main challenges”* [Participant 1]. Some also described resistance from management for allowing staff to attend half-day training sessions: *“When we were training with X NHS Trust, it was quite time consuming… I think there were three of us who were physios from the same department. The department weren’t happy letting us go as they’d be losing clinicians to training”* [Participant 9].

The challenges associated with physiotherapy appointment times and finding the opportunity within appointments to deliver brief intervention in practice were additionally discussed: *“At the moment, we have 40 minutes for a new patient, and 20 minutes for a follow up and, compared to a GP, that is a long time but when we’ve got so much to discuss… we do need longer, I think… it’s just such a rush”* [Participant 3]. These limitations were felt to be more exaggerated in the first-contact practitioner role: *“You’ve got 15 minutes to assess, diagnose, dress, undress, treat… write it all up on the computer, your time is much more limited”* [Participant 2]. For some, MECC could therefore be implemented more feasibly and effectively over a number of physiotherapy sessions: *“If you’re running behind or if a patient turns up late for an appointment, you may not have the time required to have the full conversation but it’s something I ask about in my first sessions and then save it to the next sessions to go into more depth”* [Participant 6].

#### Subtheme 2: Misperceiving MECC as being too time consuming

Others believed that time being a barrier to MECC delivery was a misperception. Rather, its principles were regarded as something that could be adapted into one’s routine interactions with patients: *“I think actually it’s a style- it shouldn’t actually take more time to do, it should just be part of the way you engage patients. That shouldn’t necessarily take any more time than doing it the direct route”* [Participant 10]. For some, practice meant that MECC skills gradually became ingrained, and were therefore no longer considered time-consuming within routine appointments: *“After a while, after people have done it a few times, you just incorporate it in your normal conversations and while you’re doing your physio stuff, you’d be having [healthy conversations] so it didn’t feel like it was taking up as much time as we first thought”* [Participant 7]. One participant shared that her own method of practice, due to the pressure of ticking boxes, had become the biggest barrier to delivering brief intervention: *“It’s so easy to say we don’t have time because we have so many things that we need to do within that short period. Sometimes you become robotic with what you need to do… and I think the main barrier is myself in terms of having that mindset, ‘Okay I have to tick all the boxes here’, you know, if you look at the principle and philosophy of MECC, you can do this within two minutes”* [Participant 8].

### Theme 5: system-level support needed to sustain MECC HCS

The final theme highlighted organisational, system-level factors for sustaining MECC HCS within physiotherapy services. Participants advocated for a MECC lead within trusts to ensure that the brief intervention is promoted, training offered, and culture change achieved. Many also felt that regular training and refreshers were important for ensuring all physiotherapists had the opportunity to develop MECC skills and that these skills could be sustained in practice.

#### Subtheme 1: a driver for MECC HCS within the trust

Most emphasised the need for a lead or champion to ensure MECC is sustained within MSK services. Without this, it was felt that training was not easily accessible to staff: *“It’s definitely supported in the trust, but I feel you’ve got to go and find it rather than it’s offered to you… I think it would very much depend on individual departments and whether you’ve got a bit of a [MECC] leader within it that runs with it”* [Participant 10]. Those that were in more senior roles discussed how they viewed themselves as being responsible for driving MECC, and contributing to culture change: *“My management role doesn’t have an indirect link in because I have oversight of staff members who I can signpost to MECC and say ‘look, this is the future, please do your training, let’s have a chat about how we can get this cultural change’. So, I’ve got my own little direct bit, doing my own little bit for my own patients, but probably the greater role I can have is actually my management role by encouraging people to take it up”* [Participant 4].

#### Subtheme 2: ongoing training and reminders

Offering annual training sessions was also considered important for sustaining MECC in practice, with many feeling they would *“benefit from a refresher”* [Participant 6]. For some, training refreshers were considered useful for ensuring healthy conversation skills were maintained: *“I think it would be worth at least being an optional training and mentioned once per year… I think there can always be more to be learnt and its always worth being reminded of questioning styles as you can always slip back into old habits when you’re in a rush”* [Participant 10]. Others emphasised a demand for training in order to raise awareness of MECC and its principles, in order to support a cultural change: *“I don’t think you would be surprised if you asked some physios what MECC is and they probably wouldn’t know what it stands for, what it is, so there is a massive need for training”* [Participant 8].

## Discussion

This study aimed to explore qualitatively the experiences and acceptability of MECC HCS for physiotherapists who support people living with MSK conditions and pain. The brief intervention was strongly supported in physiotherapy practice for several reasons.

Firstly, physiotherapists believed that MECC HCS encourages a holistic, person-centred approach to care which can enable change in patients. They felt more able to learn about patients and their contexts, and conversations regarding health and wellbeing were considered more empowering, based on the patient’s own agenda and relevant to the patient’s world. As a result, patients were felt to engage more in their own care and discussion around behaviour change. Studies have reported that patients with MSK conditions and pain regard person-centred care as highly important [51, 52]. Communicating in a way that enables patients to express their own understanding of their condition, needs and goals, has been recommended for guiding clinical interactions in physiotherapy care, allowing the patient to take an active role in the therapeutic process [53]. This can promote self-efficacy, which is associated with self-management [62]. In patients with chronic pain, it has been found that higher self-efficacy is associated with factors such as engagement in physical activity, physical functioning, disability and work status [63]. As an empowering, person-centred brief intervention that is based on Bandura’s self-efficacy [47] and underpinned by behaviour change techniques recognised to build self-efficacy, MECC HCS may therefore be promising for helping to improve the outcomes of patients with MSK conditions and pain. Patient evaluation should be considered in future research.

Secondly, participants felt that MECC HCS was highly relevant and applicable to their role as physiotherapists. Many described their routine practice as already integrating discussion around lifestyle, wellbeing, and wider determinants of health. This was felt to be an important part of care provided to patients, aligning with the recommended biopsychosocial perspective on the management of MSK conditions [64, 65]. A biopsychosocial perspective recognises pain and disability as multidimensional and dynamic, moving away from the idea of a linear association between identifiable tissue damage and pain symptoms. Rather, it is understood that biological, psychological and social factors (in addition to cognitive, physical, lifestyle etc.) interact and play a significant role in the experience of MSK conditions, pain and their persistence [66]. Psychological distress and depressive mood, for example, have been found to predict chronicity in low back pain [67], whilst social isolation and loneliness have been found to activate neural regions related to chronic pain [68–70]. It has, however, been evidenced that physiotherapists do not feel confident or competent in addressing such psychological and social factors with patients [71–73]. Our findings highlight the value of MECC HCS for facilitating a biopsychosocial approach to pain management, as participants felt that training helped to improve their competence in having holistic conversations with patients during routine practice. Interestingly, one participant highlighted how physiotherapists are generally “more biopsychosocial than other healthcare professionals”, such as her GP colleagues. In line with the critical realist approach to analysing and interpreting our qualitative data, it was important to understand the context of the individuals participating in this study and how this could influence their subjective, situated perceptions and interpretations of reality [55]. An element of social desirability may have influenced this particular response, since (a) it is recommended in clinical guidelines that physiotherapists use a biopsychosocial approach to care, and this is expected in their day-to-day practice [64] and (b) the participants had been trained in MECC HCS and were aware that it encourages a biopsychosocial approach to addressing health behaviours.

MECC HCS were additionally felt to be valuable in addressing more challenging topics relating to MSK risk factors, such as weight. This is particularly pertinent, since several studies have reported barriers faced by healthcare professionals in discussing weight management with patients, such as a fear of damage to the therapeutic relationship, and lack of confidence due to social conventions [74, 75]. Overcoming such barriers, building confidence in having these conversations early in the physiotherapy career, and embedding the relevant skills into practice was considered highly important by participants in the present study. A need for mandatory MECC HCS training at undergraduate level was therefore emphasised and is something that should be considered moving forward.

Finally, MECC HCS was highlighted as having potential to reduce pressure on health services. This pressure related to that experienced by physiotherapists themselves, the MSK services within which they work, and wider healthcare services treating MSK patients with comorbid conditions. The brief intervention was felt to promote: (a) prevention, aligning with the NHS long-term plan [76] and reducing risk of further disability and/or multimorbidity; and (b) self-management, empowering patients to independently manage their MSK conditions and pain, and having potential long-term benefits with regard to outcomes and contact with MSK services. Self-management is advocated for within MSK guidelines and policy and supports an active approach to long-term management of conditions, as opposed to passive treatments that are expected to ‘cure’ conditions, with little self-contribution [77]. Physical activity and exercise, for example, have been considered highly important, effective methods of self-managing MSK conditions [78] and can be targeted by brief intervention such as MECC HCS. Research shows that some people living with MSK conditions use physical activity to self-manage [2] and improved outcomes (i.e., reduced pain-related disability) have been evidenced [79]. However, barriers to physical activity and other self-management methods by people living with MSK conditions and pain exist [80, 81]. Moreover, multimorbidity and social deprivation can have an additional negative impact on engagement [82]. Since many people with MSK conditions are socioeconomically deprived and live with at least one other long-term condition [2], further research is warranted to understand how best to support these individuals using behavioural interventions. Considering wider determinants of health may be important for identifying and addressing their barriers to health behaviour change. Example of wider determinants of health include but are not limited to; access to education, employment, and environmental conditions. These are all factors that may impact individuals being able to or have the opportunity to, for example, learn about health risk factors and how to maintain a healthy lifestyle; afford nutritious, healthy foods and/or; access greenspaces to engage in physical activity.

The final two themes encompassed the perceptions of participants in relation to how MECC HCS could be sustained in practice, and barriers they face in implementing the brief intervention. Many highlighted time as a significant barrier to both training in and delivering MECC HCS. There appeared to be tension between perceptions of this time barrier in the short-term, and the perceived long-term positive impact that MECC HCS could have on workload pressure (as discussed above). Time as a barrier is consistent across studies that have explored the acceptability of health promotion for physiotherapists [22, 23, 83], and could be a key contributing factor to the reported missed opportunities for delivery of brief intervention during patient consultations [34, 84, 85]. It has, however, been suggested that working on changing perceptions on health promotion, so that it is seen as an integral and integrated part of all patient interactions, rather than an extra task, could increase physiotherapist engagement [22]. Interestingly, our findings showed that some physiotherapists already had this perception. MECC HCS was felt to have become ingrained into the routine practice of these individuals and a natural way of engaging with patients, taking little-to-no additional consultation time. There are thus differences in the perceived physical opportunity [24] for delivery of MECC HCS. Further work should focus on how to best support the embedding of the brief intervention into practice for those who feel that they have limited time, since according to the COM-B model of behaviour, one’s capability and motivation must interact with one’s perceived opportunity in order to achieve desired change [24].

Discussion around how best to sustain MECC HCS in practice highlighted physical opportunity [24] as a key facilitator. Participants emphasised the need for a driver for MECC HCS within departments in order to raise awareness of the brief intervention and its principles, increase accessibility to training and, ultimately, encourage a cultural change towards prevention and health promotion. Moreover, those working within trusts that did have individual MECC HCS advocates within their MSK service and/or wider organisation seemed more likely to be experiencing this shift in culture compared to those that did not. For some, buy-in from leadership was also important for driving MECC HCS and achieving this cultural change. A demand for regular training and refresher sessions was emphasised, in order to give all staff the opportunity to develop MECC HCS and ensure consistency within and across services. These findings are notable, given that MECC HCS was perceived by physiotherapists to be highly acceptable, appropriate and feasible, but only moderately sustainable, when we quantitatively explored implementation outcomes in the former stage of our sequential explanatory study [34]. These outcomes can indicate implementation success and serve as preconditions for achieving desired change [86]. Our present findings further highlight the need to increase organisational capacity for MECC HCS, ensuring that staff have enough internal support, resources and training for effective and sustained implementation. This could facilitate a cultural change towards promoting good MSK health and prevention of MSK disability in physiotherapy care, aligning with the goals of the NHS, Public Health England [76] and NICE [15, 16].

### Strengths and limitations

This study was novel in exploring the perceptions and experiences of physiotherapists delivering MECC HCS to patients living with MSK conditions and pain. We were able to gain a rich understanding of the acceptability of MECC HCS in routine physiotherapy practice, which contextualised our former, quantitative findings [34]. Using a sequential explanatory design in this way can provide an enhanced and more comprehensive answer to complex research questions than using either quantitative or qualitative methodologies alone [87]. This seems particularly pertinent when conducting applied research that aims to address practical, real-world issues. Here, we were able to assess implementation outcomes using validated, standardised, quantitative measures, whilst also exploring the contextual, subjective, and complex nature of implementation through qualitative inquiry.

However, our findings may over-represent physiotherapists who have notable experience with and positive perceptions of MECC HCS in practice. Our participants had consented to be contacted after taking part in an online survey exploring their use and perceptions of implementing MECC HCS and may have been biased towards the brief intervention and its principles. One participant, for example, had been a MECC lead within her NHS trust, and had discussed her contribution to its local rollout. Our findings and conclusions may therefore not be representative of other physiotherapists trained in MECC HCS who did not participate in this study.

### Implications for research, policy, and practice

Healthcare professionals are encouraged, during routine practice, to deliver opportunistic V/BIs and embed prevention into everyday practice. Our findings have implications in physiotherapy practice; supporting the delivery of MECC HCS with patients with MSK conditions and related pain, and meeting the goals of PHE, NHS and HEE [14, 25]. MECC HCS may encourage holistic, person-centred care, self-management and long-term prevention for this patient group which, as discussed above, may lead to improved outcomes. This is promising, as MSK conditions are a leading contributor to disability in the UK and have significant impacts on the individual, their employers and healthcare services. Our findings align with previous literature that highlight high perceived acceptability of HCS for trainees in other professions [26, 44], and suggest that MECC HCS might increase confidence and competence in having empowering conversations around health and wellbeing in routine physiotherapy practice. However, further research is warranted to explore confidence and competence further, using pre-post training and follow up outcome measures, as in other studies [88]. Patient acceptability must also be addressed, as this is currently a significant gap in the evidence base supporting MECC HCS. Findings may justify further roll out of the brief intervention in physiotherapy services. Organisational capacity for MECC HCS must, however, be addressed in order to enhance implementation and promote cultural change. Our findings suggest that physiotherapists have the motivation and capability but may not have the physical opportunity for successful implementation [24]. NHS trusts could address this by offering regular training opportunities, promoting MECC HCS through an increased number of designated champions, and endeavouring to engage senior leadership in rollout.

## Conclusion

Physiotherapist’s value MECC HCS as being integral in physiotherapy practice, promoting person-centred care, self-management and prevention in patients with MSK conditions and related pain. Physical opportunity for MECC HS must, however, be addressed in order to optimise future implementation. Further research is currently underway to explore changes in competence and confidence of physiotherapists in delivering brief interventions in practice, following MECC HCS training, and patient acceptability of its delivery during routine appointments.

### Supplementary Information

**Additional file 1.**

## Data Availability

Materials and data set are available from the corresponding author upon request.
