# Supplementary material for: ‘Making every contact count’ with patients with musculoskeletal conditions: a qualitative exploration of acceptability to physiotherapists
Source: BMC Health Serv Res. 2023 Oct 19;23:1125. doi: 10.1186/s12913-023-10126-1 (PMC10588214; doi:10.1186/s12913-023-10126-1)
Supplement: Supplementary file 1 — Additional file 1. [file 12913_2023_10126_MOESM1_ESM.docx]

**SUPPLEMENTARY FILE 1**

Semi-structured interview guide (adapted from Keyworth et al, 2019; Dewhirst and Speller, 2015).

1. If you are thinking of the patients you support on a daily basis, or those you have most regular contact with, what are the most common types of MSK conditions or complaints they present with?
2. What are the most common health behaviours you discuss with your patients?
3. How often do you identify opportunities to deliver MECC HCS to your patients?
4. How much time, if any, do you spend delivering MECC HCS in appointments with your patients?
5. To what extent do you think you are generally in a habit of ‘making every contact count’?
6. What do you think are the important or benefits, if any, of delivering MECC HCS in routine practice? For example, asking open discovery questions, listening more than giving information/ making suggestions, using SMARTER goal setting, and discussing health behaviours with patients?
7. What do you think are the importance or benefits, if any, of MECC HCS implementation to your department or organisation?
8. What do you think are the importance or benefits, if any, to your own practice or to you personally from training in and delivering MECC HCS?
9. What efforts have you noticed to promote MECC HCS at your workplace?
10. To what extent do you have the skills and confidence to engage in MECC HCS?
11. To what extent do your colleagues engage in/ support you to implement MECC HCS?
12. To what extent does your role provide the opportunity to engage in MECC HCS?
13. What are the barriers or challenges to delivering MECC HCS or supporting your patients to change their behaviours?
14. What are the barriers or challenges for patients with MSK conditions and pain themselves in changing their behaviour?
15. What are the other barriers you, your department or organisation face when implementing MECC HCS?
16. How do you record that MECC HCS has been used with an MSK service user? What is recorded? How?
17. How are referrals to other services recorded? Have there been any changes or improvements in methods of recording them?
18. How will you know if MECC HCS has had any impact?
19. What could be done to improve the recording and reviewing of MECC HCS conversations?
20. How would you improve or modify MECC HCS training to suit your needs and/or the needs of you physiotherapy team?
21. What further training do you think you or your colleague might benefit from in order to deliver MECC HCS more effectively to MSK service users?
22. What other comments do you have about this research study, the research topic or any other general comments or questions?
